# 2-Cyano-1-methyl­pyridinium iodide

**DOI:** 10.1107/S1600536813019302

**Published:** 2013-07-20

**Authors:** Michael N. Kammer, Lynn V. Koplitz, Joel T. Mague

**Affiliations:** a Department of Physics, Loyola University, New Orleans, LA 70118, USA; b Department of Chemistry, Loyola University, New Orleans, LA 70118, USA; c Department of Chemistry, Tulane University, New Orleans, LA 70118, USA

## Abstract

The cation in the title compound, C_7_H_7_N_2_^+^·I^−^, is planar (r.m.s. deviation for the nine fitted non-H atoms = 0.040 Å). The crystal packing is best described as undulating layers of cations and anions associated *via* C—H⋯I inter­actions.

## Related literature

For the structure of 2-cyano-*N*-methyl­pyridinium nitrate, see: Koplitz *et al.* (2012 ▶). For structures of 3-cyano-*N*-methyl­pyridinium salts, see: Koplitz *et al.* (2003 ▶); Mague *et al.* (2005 ▶). For structures of 4-cyano-*N*-methyl­pyridinium salts, see: Kammer, Koplitz & Mague (2012 ▶); Kammer, Mague & Koplitz (2012 ▶).
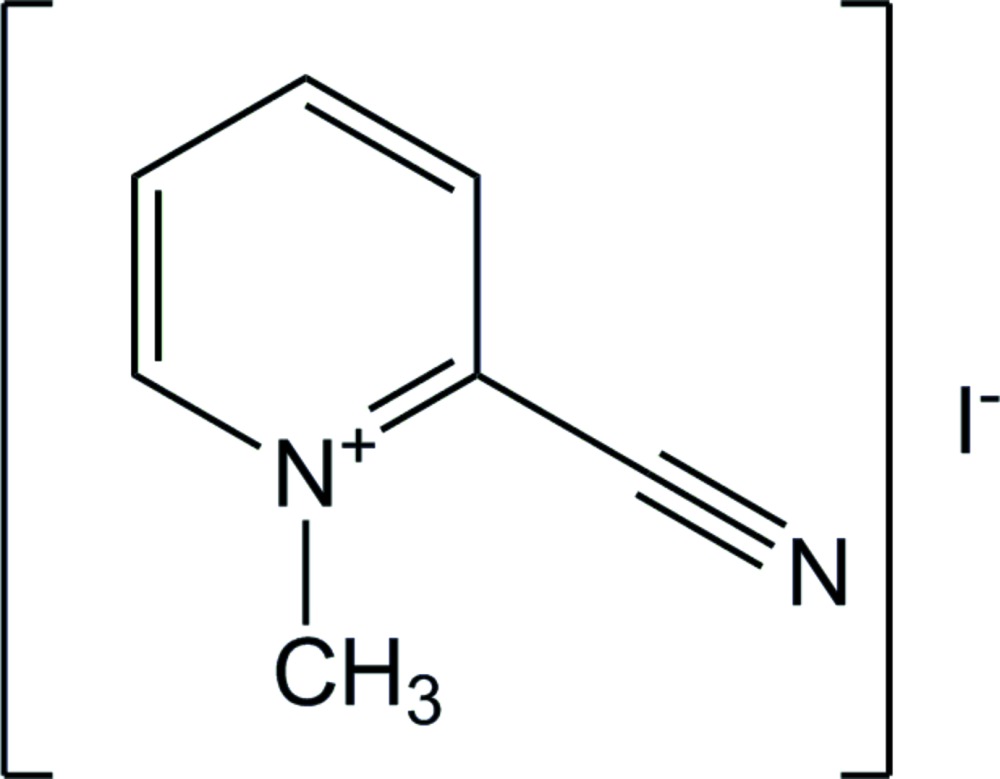


## Experimental

### 

#### Crystal data

C_7_H_7_N_2_^+^·I^−^
*M* *_r_* = 246.05
Orthorhombic, 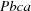

*a* = 9.5785 (6) Å
*b* = 8.5687 (5) Å
*c* = 20.2229 (13) Å
*V* = 1659.80 (18) Å^3^
*Z* = 8
Mo *K*α radiation
μ = 3.79 mm^−1^
*T* = 100 K
0.16 × 0.14 × 0.07 mm

#### Data collection

Bruker SMART APEX CCD diffractometer
Absorption correction: multi-scan (*TWINABS*; Sheldrick, 2009 ▶) *T*_min_ = 0.58, *T*_max_ = 0.77
58292 measured reflections
53390 independent reflections
40858 reflections with *I* > 2σ(*I*)
*R*_int_ = 0.047

#### Refinement

*R*[*F* ^2^ > 2σ(*F* ^2^)] = 0.036
*wR*(*F* ^2^) = 0.091
*S* = 1.03
53390 reflections
93 parameters
H-atom parameters constrained
Δρ_max_ = 1.14 e Å^−3^
Δρ_min_ = −0.43 e Å^−3^

### 

Data collection: *APEX2* (Bruker, 2013 ▶); cell refinement: *SAINT* (Bruker, 2013 ▶); data reduction: *SAINT*; program(s) used to solve structure: *SHELXTL* (Sheldrick, 2008*b* ▶); program(s) used to refine structure: *SHELXL2013* (Sheldrick, 2013 ▶); molecular graphics: *DIAMOND* (Brandenburg & Putz, 2012 ▶); software used to prepare material for publication: *SHELXTL* and *CELL_NOW* (Sheldrick, 2008*b* ▶).

## Supplementary Material

Crystal structure: contains datablock(s) global, I. DOI: 10.1107/S1600536813019302/tk5240sup1.cif

Structure factors: contains datablock(s) I. DOI: 10.1107/S1600536813019302/tk5240Isup2.hkl

Supplementary material file. DOI: 10.1107/S1600536813019302/tk5240Isup3.cml

Additional supplementary materials:  crystallographic information; 3D view; checkCIF report

## Figures and Tables

**Table 1:** Hydrogen-bond geometry (Å, °)

*D*—H⋯*A*	*D*—H	H⋯*A*	*D*⋯*A*	*D*—H⋯*A*
C1—H1*B*⋯I1^i^	0.98	3.20	4.014 (5)	141
C1—H1*C*⋯I1^ii^	0.98	3.11	4.021 (5)	156
C5—H5⋯I1^iii^	0.95	3.12	3.810 (5)	131
C6—H6⋯I1^iv^	0.95	3.03	3.677 (5)	126

Symmetry codes: (i) 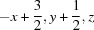; (ii) 
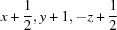
; (iii) 
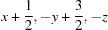
; (iv) 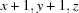.

**Fig. 1.**
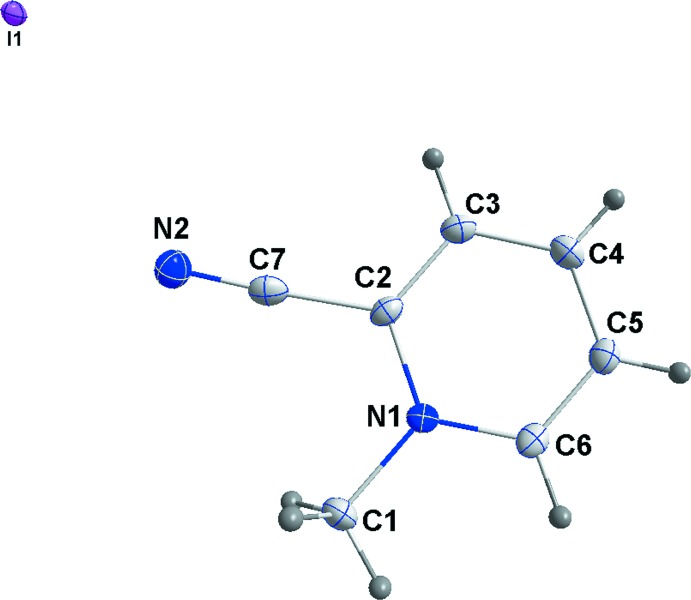
Perspective view of the title compound with 50% probability ellipsoids.

**Fig. 2.**
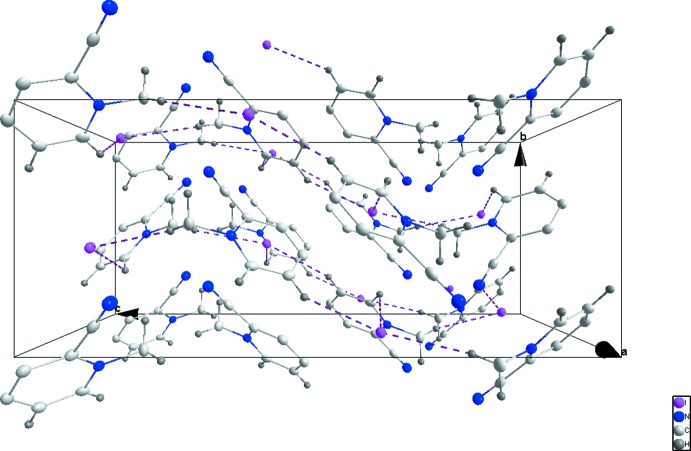
Packing projected down a showing the side view of the layers.

**Fig. 3.**
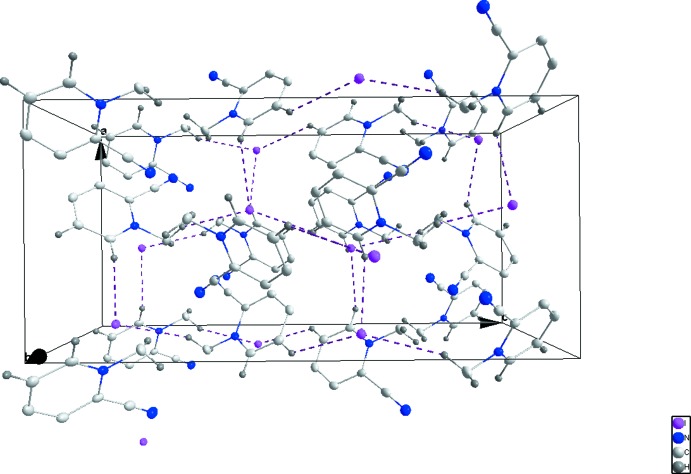
Packing projected down b showing the top view of the layers.

## supplementary crystallographic information

### Comment

The three-dimensional, solid state structures of the salts of the three isomeric cyano-*N*-methylpyridinium cations are quite varied. These range from layered structures as seen in the chloride and bromide salts of the 3-cyano-*N*-methyl pyridinium cation (Koplitz, *et al.*, 2003; Mague, *et al.*, 2005) and in 2-cyano-N– methylpyridinium nitrate (Koplitz, *et al.*, 2012) through an open channel structure for 3-cyano-*N*-methyl pyridinium iodide (Koplitz, *et al.*, 2003) to complex networks as found for 4-cyano-*N*-methylpyridinium bromide and iodide (Kammer, Mague & Koplitz, 2012; Kammer, Koplitz & Mague, 2012). In all instances, the packing appears to be organized by weak C—H to anion hydrogen bonding and, in the case of 2-cyano-*N*-methylpyridinium nitrate, an anion–π interaction although we have yet to discern a pattern based on either the position of the cyano group on the ring or the size or shape of the anion. In the title compound, the packing of the cations and anions is organized by weak C—H···I hydrogen bonding (Table 1). It can be described as "wavy" layers extending parallel to *c*. Fig. 2 presents a side view of the layers while Fig. 3 is a top view. From both of these, it is evident that the weak interionic interactions organize a three-dimensional network which is intermediate in complexity between those seen for 3-cyano-*N*-methylpyridinium iodide and 4-cyano-*N*-methylpyridinium bromide.

### Experimental

2-Cyanopyridine (10.5 g) was first melted in a warm water bath and then dissolved in benzene (40 ml). Iodomethane (9.5 ml) was added to this solution slowly with stirring and the solution was refluxed for 2 h. Yellow solid 2-cyano-*N*-methyl pyridinium iodide (m.p. 419–423 K) was collected by vacuum filtration.

### Refinement

The diffraction data were obtained from 3 sets of 400 frames, each of width 0.5° in ω, collected at φ = 0.00, 90.00 and 180.00° and 2 sets of 800 frames, each of width 0.45° in φ, collected at ω = -30.00 and 210.00°. The scan time was 15 sec/frame. Analysis of 1649 reflections having I/σ(I) > 15 and chosen from the full data set with *CELL_NOW* (Sheldrick, 2008*a*) showed the crystal to belong to the orthorhombic system and to be twinned by a 180 ° rotation about c. The raw data were processed using the multi-component version of *SAINT* under control of the two-component orientation file generated by *CELL_NOW*. The model was refined as a two-component twin which, although giving somewhat higher values for R1 and wR2 and the residual peaks in the final difference map than refinement as 1-component using the single component reflection file extracted from the full data set with TWINABS, provided a more reasonable suggested weighting scheme. H-atoms attached to carbon were placed in calculated positions (C—H = 0.95 - 0.98 Å). All were included as riding contributions with isotropic displacement parameters 1.2 - 1.5 times those of the attached atoms. The maximum and minimum residual electron density peaks of 1.14 and 0.43 eÅ^-3^, respectively, were located 0.02 Å and 1.86 Å from the I1 and N1 atoms, respectively.

### Crystal data

**Table:**

C_7_H_7_N_2_^+^·I^−^	*D*_x_ = 1.969 Mg m^−^^3^
*M**_r_* = 246.05	Melting point: 146 K
Orthorhombic, *P**b**c**a*	Mo *K*α radiation, λ = 0.71073 Å
*a* = 9.5785 (6) Å	Cell parameters from 4287 reflections
*b* = 8.5687 (5) Å	θ = 2.9–28.7°
*c* = 20.2229 (13) Å	µ = 3.79 mm^−^^1^
*V* = 1659.80 (18) Å^3^	*T* = 100 K
*Z* = 8	Slab, yellow
*F*(000) = 928	0.16 × 0.14 × 0.07 mm

### Data collection

**Table:**

Bruker SMART APEX CCD diffractometer	53390 independent reflections
Radiation source: fine-focus sealed tube	40858 reflections with *I* > 2σ(*I*)
Graphite monochromator	*R*_int_ = 0.047
Detector resolution: 8.3660 pixels mm^-1^	θ_max_ = 28.7°, θ_min_ = 2.0°
φ and ω scans	*h* = −12→12
Absorption correction: multi-scan (*TWINABS*; Sheldrick, 2009)	*k* = −11→11
*T*_min_ = 0.58, *T*_max_ = 0.77	*l* = −26→27
58292 measured reflections	

### Refinement

**Table:**

Refinement on *F*^2^	Primary atom site location: structure-invariant direct methods
Least-squares matrix: full	Secondary atom site location: difference Fourier map
*R*[*F*^2^ > 2σ(*F*^2^)] = 0.036	Hydrogen site location: inferred from neighbouring sites
*wR*(*F*^2^) = 0.091	H-atom parameters constrained
*S* = 1.03	*w* = 1/[σ^2^(*F*_o_^2^) + (0.0143*P*)^2^ + 0.7747*P*] where *P* = (*F*_o_^2^ + 2*F*_c_^2^)/3
53390 reflections	(Δ/σ)_max_ < 0.001
93 parameters	Δρ_max_ = 1.14 e Å^−^^3^
0 restraints	Δρ_min_ = −0.43 e Å^−^^3^

### Special details

**Table:**

Geometry. All e.s.d.'s (except the e.s.d. in the dihedral angle between two l.s. planes) are estimated using the full covariance matrix. The cell e.s.d.'s are taken into account individually in the estimation of e.s.d.'s in distances, angles and torsion angles; correlations between e.s.d.'s in cell parameters are only used when they are defined by crystal symmetry. An approximate (isotropic) treatment of cell e.s.d.'s is used for estimating e.s.d.'s involving l.s. planes.

### Fractional atomic coordinates and isotropic or equivalent isotropic displacement parameters (Å^2^)

**Table:**

	*x*	*y*	*z*	*U*_iso_*/*U*_eq_	
I1	0.41161 (3)	0.07802 (3)	0.10870 (2)	0.01550 (12)	
N1	0.9765 (4)	1.0275 (5)	0.1442 (2)	0.0135 (9)	
N2	0.7422 (4)	0.7530 (5)	0.2022 (2)	0.0205 (10)	
C1	1.0446 (5)	0.9818 (6)	0.2072 (3)	0.0187 (11)	
H1A	1.1337	1.0371	0.2116	0.028*	
H1B	1.0615	0.8690	0.2072	0.028*	
H1C	0.9837	1.0092	0.2443	0.028*	
C2	0.8562 (5)	0.9559 (6)	0.1244 (3)	0.0142 (10)	
C3	0.7917 (5)	0.9967 (6)	0.0661 (3)	0.0158 (11)	
H3	0.7074	0.9471	0.0530	0.019*	
C4	0.8517 (5)	1.1118 (6)	0.0268 (3)	0.0168 (11)	
H4	0.8105	1.1395	−0.0143	0.020*	
C5	0.9713 (5)	1.1848 (6)	0.0481 (3)	0.0166 (11)	
H5	1.0122	1.2648	0.0220	0.020*	
C6	1.0321 (5)	1.1424 (5)	0.1070 (3)	0.0149 (10)	
H6	1.1140	1.1945	0.1216	0.018*	
C7	0.7957 (5)	0.8415 (6)	0.1685 (3)	0.0165 (11)	

### Atomic displacement parameters (Å^2^)

**Table:**

	*U*^11^	*U*^22^	*U*^33^	*U*^12^	*U*^13^	*U*^23^
I1	0.01513 (19)	0.01697 (18)	0.01439 (19)	0.00131 (12)	−0.00021 (13)	−0.00058 (13)
N1	0.013 (2)	0.014 (2)	0.014 (2)	0.0017 (16)	0.0001 (18)	−0.0019 (17)
N2	0.022 (2)	0.020 (2)	0.019 (2)	−0.0019 (19)	0.001 (2)	−0.0010 (19)
C1	0.020 (3)	0.022 (3)	0.014 (3)	−0.001 (2)	−0.004 (2)	0.001 (2)
C2	0.012 (2)	0.014 (2)	0.016 (3)	−0.0010 (19)	0.003 (2)	−0.003 (2)
C3	0.013 (3)	0.016 (2)	0.018 (3)	0.0006 (19)	0.000 (2)	−0.003 (2)
C4	0.019 (3)	0.017 (2)	0.014 (3)	0.004 (2)	−0.001 (2)	−0.002 (2)
C5	0.017 (3)	0.014 (2)	0.020 (3)	0.0014 (19)	0.003 (2)	0.000 (2)
C6	0.012 (2)	0.011 (2)	0.021 (3)	0.0023 (18)	0.003 (2)	−0.002 (2)
C7	0.015 (2)	0.019 (3)	0.016 (3)	0.001 (2)	−0.001 (2)	−0.006 (2)

### Geometric parameters (Å, º)

**Table:**

N1—C6	1.348 (6)	C2—C7	1.446 (7)
N1—C2	1.366 (6)	C3—C4	1.391 (7)
N1—C1	1.484 (6)	C3—H3	0.9500
N2—C7	1.141 (6)	C4—C5	1.375 (7)
C1—H1A	0.9800	C4—H4	0.9500
C1—H1B	0.9800	C5—C6	1.375 (7)
C1—H1C	0.9800	C5—H5	0.9500
C2—C3	1.375 (7)	C6—H6	0.9500
			
C6—N1—C2	119.8 (4)	C2—C3—H3	120.5
C6—N1—C1	119.9 (4)	C4—C3—H3	120.5
C2—N1—C1	120.3 (4)	C5—C4—C3	119.1 (5)
N1—C1—H1A	109.5	C5—C4—H4	120.4
N1—C1—H1B	109.5	C3—C4—H4	120.4
H1A—C1—H1B	109.5	C6—C5—C4	120.3 (5)
N1—C1—H1C	109.5	C6—C5—H5	119.8
H1A—C1—H1C	109.5	C4—C5—H5	119.8
H1B—C1—H1C	109.5	N1—C6—C5	120.6 (5)
N1—C2—C3	121.1 (4)	N1—C6—H6	119.7
N1—C2—C7	117.5 (4)	C5—C6—H6	119.7
C3—C2—C7	121.4 (5)	N2—C7—C2	176.9 (6)
C2—C3—C4	119.0 (5)		
			
C6—N1—C2—C3	1.3 (7)	C2—C3—C4—C5	−2.0 (7)
C1—N1—C2—C3	−180.0 (5)	C3—C4—C5—C6	1.2 (7)
C6—N1—C2—C7	−175.6 (4)	C2—N1—C6—C5	−2.2 (7)
C1—N1—C2—C7	3.1 (7)	C1—N1—C6—C5	179.1 (4)
N1—C2—C3—C4	0.7 (7)	C4—C5—C6—N1	0.9 (7)
C7—C2—C3—C4	177.5 (5)		

### Hydrogen-bond geometry (Å, º)

**Table:**

*D*—H···*A*	*D*—H	H···*A*	*D*···*A*	*D*—H···*A*
C1—H1*B*···I1^i^	0.98	3.20	4.014 (5)	141
C1—H1*C*···I1^ii^	0.98	3.11	4.021 (5)	156
C5—H5···I1^iii^	0.95	3.12	3.810 (5)	131
C6—H6···I1^iv^	0.95	3.03	3.677 (5)	126

Symmetry codes: (i) −*x*+3/2, *y*+1/2, *z*; (ii) *x*+1/2, *y*+1, −*z*+1/2; (iii) *x*+1/2, −*y*+3/2, −*z*; (iv) *x*+1, *y*+1, *z*.
